# Maxillary foreign body management in odontogenic sinusitis: an algorithm based on a single centre experience

**DOI:** 10.3389/froh.2026.1945885

**Published:** 2026-10-14

**Authors:** Lorenzo Sabatino, Michele Antonio Lopez, Matteo Valentini, Marco Mattaroccia, Francesco Iafrati, Simone Di Giovanni, Antonio Moffa, Manuele Casale

**Affiliations:** 1 Fondazione Policlinico Universitario Campus Bio-Medico, Rome, Italy; 2 Fondazione Policlinico Universitario Agostino Gemelli IRCCS, Rome, Italy; 3 Universita Campus Bio-Medico di Roma, Rome, Italy

**Keywords:** endoscopic middle meatus antrostomy, maxillary sinus foreign body, modified mini Caldwell-Luc, multidisciplinary surgery, odontogenic sinusitis, treatment algorithm

## Abstract

**Purpose:**

This study aimed to evaluate the management of patients with odontogenic maxillary sinusitis associated with foreign bodies.

**Methods:**

We retrospectively reviewed 165 patients treated at our institution; 48 met the inclusion criteria and were analyzed. All presented odontogenic sinusitis with a maxillary sinus foreign body and underwent surgery according to a multidisciplinary treatment algorithm based on clinical and radiological findings.

**Results:**

Thirty-two patients were treated with a simultaneous combined oral and nasal approach, 12 with an oral approach alone, and 4 with a nasal approach alone. Complete removal of the foreign body and complete symptom resolution were achieved in all cases.

**Conclusion:**

In this retrospective series, surgical treatment was successful in all 48 patients. Our results support that a multidisciplinary approach was the most frequently utilized strategy in our cohort and proved highly effective in appropriately selected patients for the management of odontogenic sinusitis associated with maxillary foreign bodies, allowing surgeons to tailor the surgical approach to the specific anatomical and clinical presentation.

## 1 Introduction

According to the EPOS 2020 classification, odontogenic sinusitis is classified as secondary chronic rhinosinusitis, similar to other phenotypes such as neoplasms or fungus ball, and is therefore regarded as a localized disease. It has an anatomic unilateral distribution (1). Although sinusitis predominantly originates from rhinogenic sources, dental infections may represent significant predisposing factors in a subset of cases (2).

The primary and widely recognized cause of odontogenic sinusitis (ODS) is periapical or periodontal infection originating from a maxillary posterior tooth, in which inflammatory exudate erodes through the bone and drains into the sinus (3).

Hoskinson reported that periapical lesions accounted for 73% of odontogenic sinusitis (ODS) cases, oroantral fistulae for 23%, and retained teeth for 4% (4). Even if bilateral involvement is documented, odontogenic sinusitis (ODS) is typically suspected only in cases of unilateral rhinosinusitis accompanied by chronic maxillary dental issues or previous dental procedures in that anatomical region (5). Odontogenic sinusitis (ODS) develops unilaterally in over 90% of cases, with 85% to 90% of patients experiencing ipsilateral sinonasal symptoms (6). Odontogenic sinusitis predominantly affects individuals aged 40 to 60 years, with a slight predominance in the female sex. Approximately half of the patients report a history of prior dental surgery or infection, although only about one-third experience associated dental pain. The clinical manifestations of odontogenic sinusitis are variable; however, the most frequent symptoms include facial pain or pressure, postnasal drip, nasal obstruction, and unilateral purulent anterior rhinorrhea often accompanied by a foul odor or taste and general fatigue. Furthermore, anterior rhinoscopy or nasal endoscopy may reveal unilateral purulent discharge or mucosal edema, though these examinations are less sensitive for diagnosing odontogenic sinusitis compared with radiologic imaging techniques. It's important to note that dental pain is frequently absent in cases of odontogenic sinusitis. Moreover, when dental pain occurs without accompanying nasal symptoms, it is not a definitive indicator of sinusitis (7, 8).

A multidisciplinary approach is required to diagnose odontogenic sinusitis. A diagnostic suspicion of odontogenic sinusitis should arise in the presence of unilateral maxillary sinus opacification on CT, evidence of dental pathology on imaging, endoscopic findings such as unilateral middle meatal purulence, a foul odor, or the identification of odontogenic bacteria on sinus culture. A multidisciplinary approach is essential to achieve a prompt and accurate diagnosis. Otolaryngologic assessment is required to confirm sinus involvement through endoscopic evaluation, which may reveal purulent discharge, mucosal edema, or polyps; if endoscopic findings are negative, the diagnosis may rely on symptoms or a positive CT scan. Simultaneously, oral surgical consultation is equally critical to confirm the odontogenic origin through intraoral examination, assessment of dental implants for peri-implantitis or mobility, and imaging studies such as CBCT, orthopantomography, or periapical radiography (9).

Numerous studies report partial to complete maxillary sinus opacification in 70%–100% of odontogenic sinusitis (ODS) cases. Isolated maxillary sinus mucosal thickening is highly prevalent (45%–100%) among individuals with adjacent maxillary dental pathology or prior dental procedures (10). Otolaryngologists must evaluate the maxillary dentition on sinus CT, as dental pathology is reportedly overlooked in 60%–70% of cases by radiologists. Although patients with odontogenic sinusitis may exhibit sinus CT findings with obvious dental disease, such as oroantral fistulae or periapical lesions associated with alveolar bone loss, these pathologies are not consistently recognized without targeted dental assessment (11). The larger the periapical lesion diameter and the closer it is to the maxillary sinus floor, the more pronounced the maxillary sinus opacification and ostiomeatal complex obstruction tend to be (12).

Odontogenic sinusitis commonly arises when the mucoperiosteum or Schneiderian membrane of the maxillary sinus is compromised. Odontogenic sinusitis related to dental implants commonly arises following dental interventions including maxillary implant placement, sinus lift procedures, perforation of the Schneiderian membrane of the maxillary sinus, and migration of implants or augmentation materials into the sinus space (13).

While mucositis is defined as an inflammation of the peri-implant mucosa without bone loss, peri-implantitis is characterized by inflammatory signs of peri-implant tissues and bone loss after functional marginal bone remodeling (14).

Primarily, accidental displacements of dental implants into the maxillary sinuses occur intraoperatively, often related to excessive implant site preparation, insufficient primary stability, or inadequate surgical planning. In these cases, the displacement happens as a surgical complication, where the implant inadvertently moves through the maxillary sinus during the placement procedure. Conversely, implant migration into the maxillary sinus can also occur years after placement, although the exact cause of this delayed migration remains unclear. Potential mechanisms proposed include inflammatory responses such as peri-implantitis or bone resorption due to improper occlusal load distribution (15). These foreign bodies found in the odontogenic maxillary sinus vary in composition and include tooth roots, root canal filling materials, fractured tooth fragments, dental implants, and dental impression substances. Entry of these foreign objects into the maxillary sinus can occur through several pathways, such as apical migration of filling materials, inadvertent traumatic manipulation during dental procedures, or via an oroantral fistula (16).

An entire tooth may be displaced into the maxillary sinus, particularly during third molar extraction. Conservative retrieval through the socket is initially indicated; if no sinus membrane perforation is evident and the root fragment measures ≤3 mm without infection, it may be left *in situ* to minimize patient morbidity, with standard sinus precautions, decongestants, and antibiotics prescribed. Primary retrieval is warranted for infected root fragments or those exceeding 3 mm in size (17).

The resorption of the alveolar ridge together with progressive pneumatization of the maxillary sinus leads to a reduction in the vertical dimension of the edentulous posterior maxilla. Insufficient residual bone height is a major predisposing factor for implant displacement in this region and represents a contraindication to implant placement (18).

The aim of this study is to retrospectively analyze the experience of the department of integrated therapies in otolaryngology (Campus Bio-Medico Foundation, Rome, Italy) in treating patients affected by odontogenic sinusitis with foreign body, in order to identify the best surgical approach in each subset of patients.

## 2 Materials and methods

A retrospective analysis was conducted on the clinical records of patients who underwent surgical treatment for odontogenic sinusitis associated with the presence of a foreign body at the Department of Integrated Therapies in Otolaryngology, Campus Bio-Medico University, Rome, Italy, between December 2020 and November 2025.

Data collected included demographic characteristics, etiological factors, and therapeutic management. Patients were selected based on the following inclusion criteria: (1) a clinical diagnosis of sinusitis of suspected odontogenic origin, by making an etiological distinction between acute and chronic sinusitis, as determined by a three-month time criterion, supported by radiological and/or endoscopic evidence and unresponsive to medical therapy; (2) the presence of a foreign body associated with sinusitis; and (3) the availability of preoperative computed tomography (CT) imaging.

The diagnosis of sinusitis was established in accordance with the EPOS 2020 guidelines, whereby characteristic signs and symptoms of the condition such as purulent rhinorrhea (anterior and/or posterior), unilateral or bilateral nasal obstruction, and maxillary pain were observed. These clinical features were corroborated by endoscopic examination and/or computed tomography (CT) imaging, in conjunction with the presence of a foreign body, and demonstrated resistance to medical therapy, which typically consisted of topical nasal decongestants or corticosteroids, mucolytic agents, and systemic antibiotics.

From a radiological point of view, a maxillofacial computed tomography (CT) scan and a dental scan were recommended for all patients to assess maxillary sinus involvement and to evaluate the nasal and paranasal sinus anatomy for optimal preoperative treatment planning.

Patients were further evaluated based on other clinical conditions, such as the presence or absence of an oroantral fistula or communication, and OMC obstruction or a free OMC as determined by the CT scan.

Furthermore, the classification system proposed by Felisati et al. was applied to categorize patients into three groups: pre-implantological complications, implantological complications, and classical complications. Based on these parameters, a multidisciplinary evaluation was performed to determine the tailored surgical management for the foreign body removal.

### 2.1 Bias assessment

To minimize measurement and observer bias, all diagnostic modalities were subjected to a rigorous multidisciplinary review. Specifically, preoperative computed tomography (CT) scans were independently assessed by at least three specialists (a radiologist, an otolaryngologist, and an oral surgeon), while preoperative and postoperative endoscopic findings were evaluated jointly by at least two specialists (an otolaryngologist and an oral surgeon).

Postoperative follow-up consisted of clinical and endoscopic evaluations. The minimum required follow-up duration to confirm complete symptom resolution was 3 months.

### 2.2 Surgical techniques

#### 2.2.1 Nasal approach: endoscopic middle meatus antrostomy (MMA)

The surgical steps related to endoscopic middle meatus antrostomy (MMA) included an uncinectomy and the creation of a middle meatal antrostomy to open the maxillary sinus, followed by the thorough clearance of pathological mucus or tissue. The natural sinus ostium was surgically enlarged, preserving the healthy sinus mucosa and its basement membrane to maintain mucociliary function. The width of the ostium depended on the extent of the pathological involvement. A widely patent ostium can also serve as an access route for the removal of the foreign body (Figure 1). Due to the close proximity of critical anatomical structures, including the orbit and the infraorbital nerve, the procedure requires advanced surgical expertise and precision. The approach seeks to restore natural drainage while minimizing tissue damage and postoperative complications.

**Figure 1:**
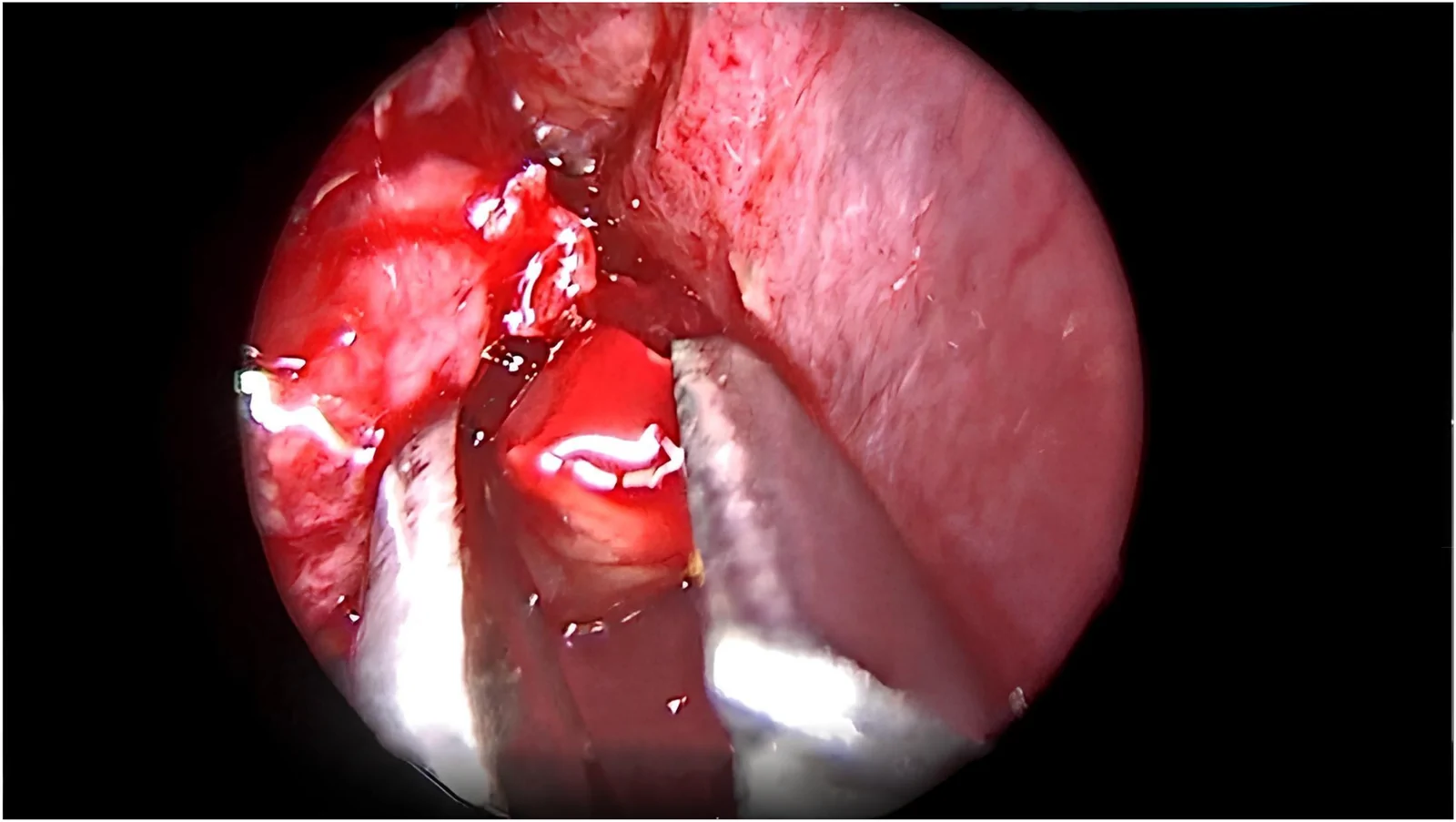
Foreign body removal from maxillary sinus after a middle meatal antrostomy.

### 2.3 Oral approach: modified mini Caldwell-Luc

A full-thickness mucoperiosteal triangular or rectangular flap is elevated from teeth 3 to 6 (canine to first molar), corresponding to the canine fossa. Dissection is carried out until the canine fossa is completely exposed. The cranial margin of the flap is determined by the course of the infraorbital artery, the mesial margin should be as anterior as possible, the caudal margin is placed approximately 3 mm superior to the sinus floor, and the distal margin is adapted according to the number of planned implants. Depending on the position and size of the foreign body, the flap can be extended more cranially if necessary. A bony window is then created in the anterior wall of the maxillary sinus using a bur until the Schneiderian membrane is exposed. The membrane is carefully incised to gain access to the sinus cavity.

The size of the antrostomy is adapted to the foreign body dimensions and typically exceeds 3 to 4 mm to allow the introduction of a 2.5 to 3 mm pediatric endoscope for visualization and subsequent removal of the foreign body (Figure 2).

**Figure 2:**
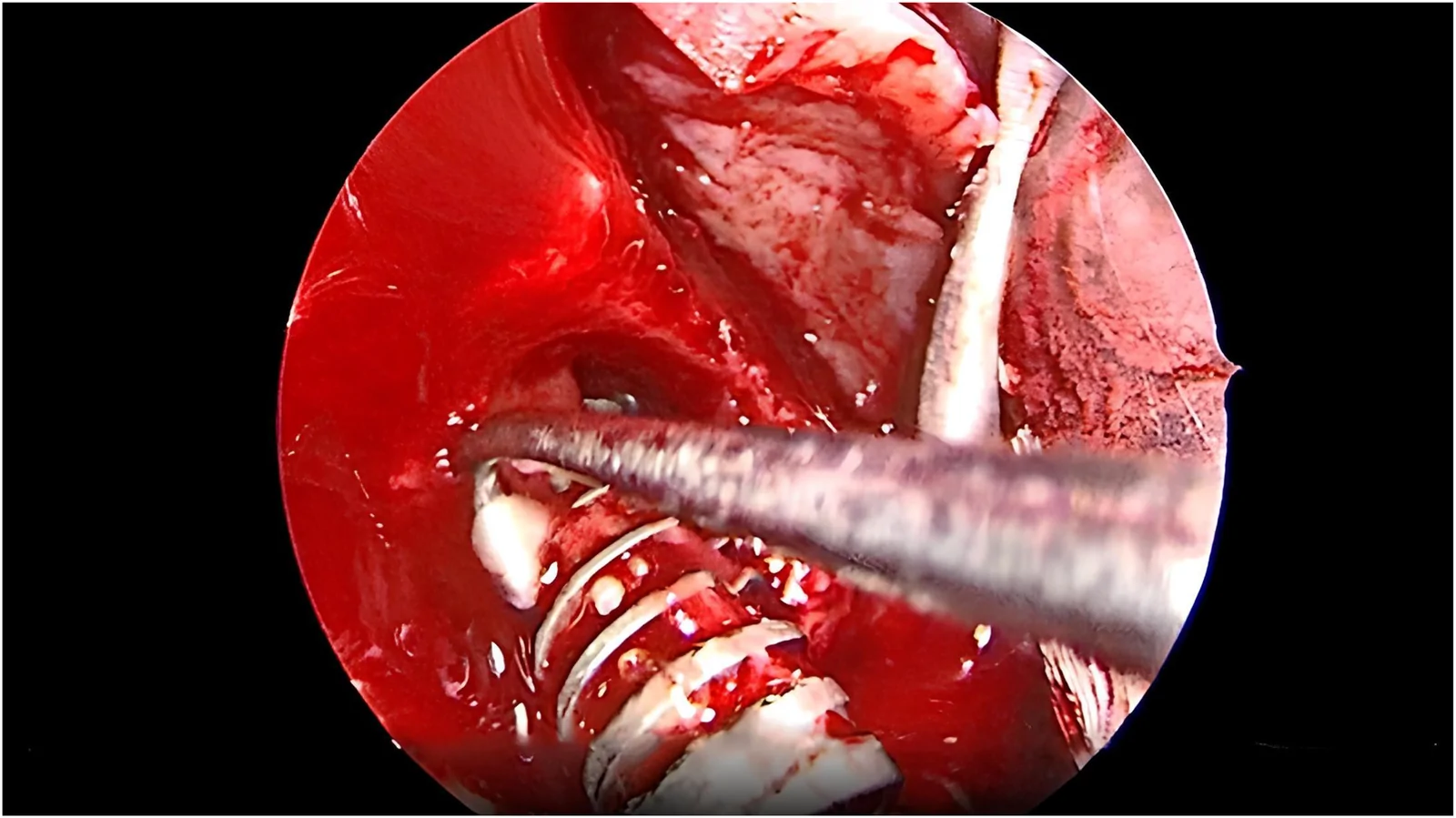
Foreign body removal via modified mini Caldwell-Luc with canine fossa antrostomy.

Final closure was achieved by repositioning the flap and securing it with sutures.

This modification reduces postoperative morbidity such as facial swelling and paresthesia while facilitating effective sinus clearance with quicker recovery compared to the traditional Caldwell-Luc procedure.

### 2.4 Surgical approaches for OAC/OAF closure

In case of the presence of an OAC/OAF of adequate size compared to the size of the foreign body, it can be used as a route for the foreign body removal.

After removal, the different techniques for the closure of OAC/OAF have been described in our previous retrospective clinical study:

Local flap technique: uses nearby soft tissue to close small to medium defects (between 5 and 8 mm) with primary suture closure.

Cortico-cancellous graft with a resorbable collagen membrane: involves elevating a full-thickness flap to access the sinus, implanting a bone graft, and covering it with a collagen membrane to enhance bone regeneration in larger defects with sufficient adjacent bone (defects larger than 8 mm with sufficient adjacent bone vestibularly and palatally to the defect)

Technique with a Cortico-Cancellous Graft Covered with a Resorbable Collagen Membrane and a Heterologous Cortical Lamina: used for defects larger than 8 mm with reduced adjacent bone. A rigid cortical lamina oversized by about 2 mm covers a heterologous cortico-cancellous graft, stabilized with thermoplastic gel or pins to enhance support and promote bone regeneration (19).

### 2.5 Statistical analysis

Given the specific aims of the study and the sample size constraints within certain surgical subgroups (e.g., only four patients in the nasal-only group), no formal comparative superiority analyses were performed. The study is intentionally presented as a descriptive retrospective case series. The primary objective was to descriptively report the utilization and clinical outcomes of the proposed treatment algorithm rather than to prove statistical superiority among the surgical techniques.

### 2.6 Ethics statement

The study was conducted in accordance with the Declaration of Helsinki. Ethical review and approval were waived for this study due to its retrospective observational nature, based exclusively on the analysis of pre-existing routine clinical data.

## 3 Foreign body removal techniques

In this study, although we recognize that an oroantral communication (OAC, acute breach) and an oroantral fistula (OAF, chronic epithelialized tract) are distinct clinical entities, they were grouped together as “oroantral defects” (OAC/OAF) for the purposes of our treatment algorithms, as their surgical management and the need for closure overlap substantially. We adopted different surgical techniques to optimally manage patients presenting with foreign bodies, taking into account the presence and dimensions of the oroantral communication/fistula. Several other parameters guided the choice of the most appropriate approach, including the size and location of the foreign body. The condition of the ostio-meatal complex (OMC) also played a key role in determining the surgical strategy, distinguishing the patients affected and unaffected by odontogenic sinusitis.

- Fistular approach: Presence of an oroantral fistula with a foreign body whose size is smaller than the fistulous tract. Therefore, the foreign body is removed through the fistulous tract.
- Nasal approach (MMA): Removal of the foreign body through a middle meatal antrostomy.
- Oral approach (Modified Mini Caldwell-Luc): Removal of the foreign body via an oral approach.

## 4 Foreign body approaches depending on different phenotypical presentations

Taking into account the clinical presentation, the presence or absence of sinusitis the existence of an OAC/OAF, and the position and size of the foreign body, we adopted different approaches, tailoring the combination of the above-mentioned techniques to each case. We therefore analyzed the various scenarios according to the changes in these phenotypic variables.

In case of:

4.1 Patients without OMC obstruction, with the absence of an OAC/OAF and a foreign body dimension less than 1 to 2 cm, we adopted a nasal approach or a lateral approach *depending on the foreign body position*. For foreign bodies located inferiorly or embedded in inflammatory tissue within the deep recesses of the maxillary sinus, an oral approach is preferred (mini Caldwell-Luc technique). Conversely, when the foreign body is positioned superiorly or adherent to the roof/upper recess of the sinus, a nasal endoscopic approach (MMA or its variants) is more suitable, enabling direct and safe access to the upper sinus portion*.*
4.2 Patients without OMC obstruction, with no OAC/OAF and a foreign body dimension larger than 2 cm, an endoscopically assisted lateral approach was our surgical choice, since it would be difficult to remove large foreign bodies from the middle meatus.
4.3 Patients unaffected by odontogenic sinusitis, with the presence of an OAC/OAF and a foreign body dimension less than 1 to 2 cm, we performed a fistular approach (Sabatino et al.) associated with fistula closure. In this case it was possible to perform a fistular approach according to the foreign body size.
4.4 Patients unaffected by odontogenic sinusitis, in the context of an OAC/OAF and a foreign body dimension larger than 2 cm, a lateral approach associated with fistula closure or a fistular approach was performed. The choice was made in favor of one rather than the other depending on the FB size. The latter occurred in case the FB dimension was smaller than that of the OAF.

Our personal revision of the flowchart of foreign body management in patients without signs and symptoms of sinusitis from:

- A. González-García at al. (15)
- Brescia G. et al. (28) (Figure 3)
4.5 Patients affected by OMC obstruction, with the absence of an OAC/OAF and a foreign body dimension smaller than 1 to 2 cm, a combined approach or the nasal approach alone were the surgical strategies. Thus, both the *size* and especially the *location* of the foreign body often make an exclusively nasal approach insufficient for proper management, as it would require excessive enlargement of the medial wall of the maxillary sinus, particularly at the level of the natural ostium.
4.6 Patients affected by odontogenic sinusitis (OMC obstruction), with no OAC/OAF and a foreign body dimension larger than 2 cm were treated with a combined approach, consisting of a simultaneous endoscopic nasal and lateral approach (mini Caldwell-Luc).
4.7 Patients affected by odontogenic sinusitis (OMC obstruction), with coexistence of an OAC/OAF and a foreign body dimension smaller than 1 to 2 cm, a combined approach was performed. In particular, this involved a maxillary antrostomy and the nasal removal of the foreign body, followed by a fistular approach in order to obtain an OAF closure.
4.8 Patients affected by odontogenic sinusitis (OMC obstruction), with the presence of an OAC/OAF and a foreign body dimension larger than 2 cm, the surgical choice fell on the combined approach. This surgical procedure consisted of a middle meatal antrostomy (MMA) and foreign body removal via a fistular or lateral approach.

**Figure 3:**
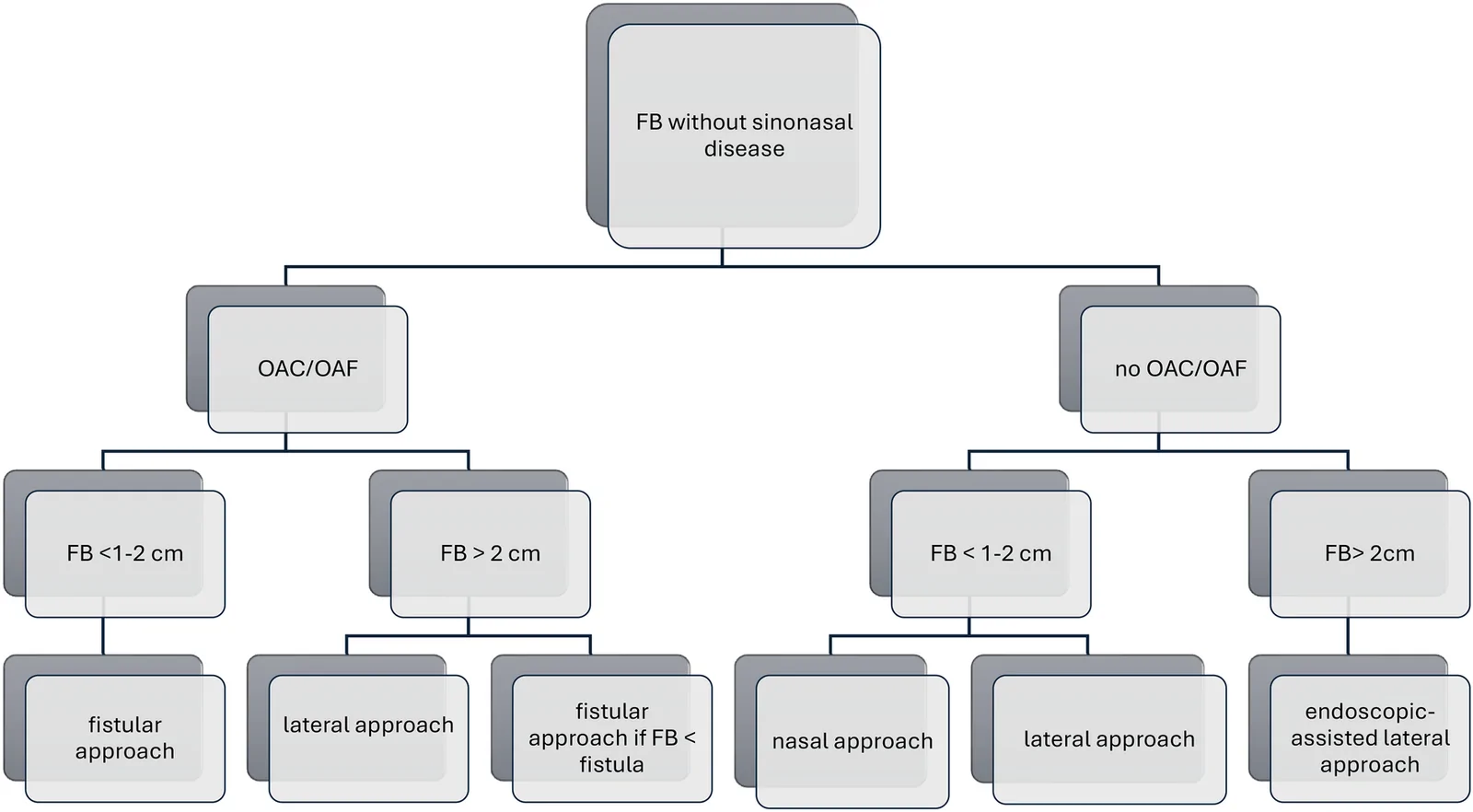
Algorithm for the management of foreign bodies in patients without odontogenic sinusitis.

It is appropriate to highlight one of the limitations of the nasal approach; it could be insufficient for adequate management and would necessitate an excessive enlargement of the medial wall of the maxillary sinus, particularly at the level of the natural ostium. This specific concurrency of surgical techniques was performed when the foreign body exceeded 2 cm and was positioned distant from the natural maxillary ostium, after which fistula closure was performed. A fundamental aspect highlighted in this clinical condition is the combined approach, which emphasizes the critical importance of a multidisciplinary collaboration between the otolaryngologist and the dentist, aiming to determine the most suitable surgical route. The characteristics of the foreign body, along with the presence or absence of signs and symptoms of sinusitis have therefore led us to perform, in the vast majority of cases, to opt for the simultaneous use of a combined approach.

Our personal revision of the flowcharts of foreign body management in patients with signs and symptoms of odontogenic sinusitis from:

- A. González-García at al. (15)
- Brescia G. et al. (28) (Figure 4)

**Figure 4:**
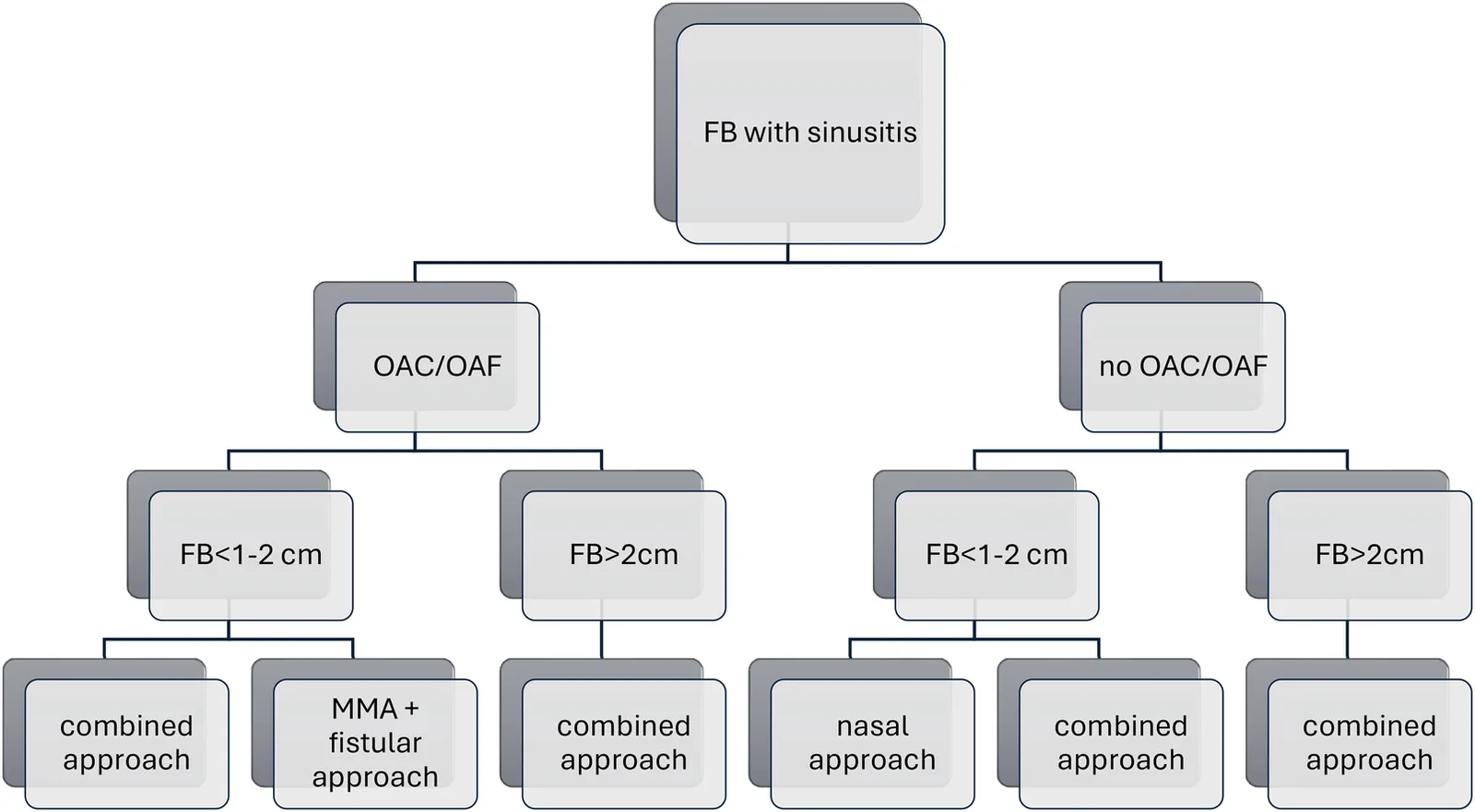
Algorithm for the management of foreign bodies in patients with odontogenic sinusitis.

Oral approaches:

The oral approach was performed considering the presence or absence of an oro-antral communication or fistula, with particular attention to the position and especially the size of the foreign body.

- An endoscopic oral technique was applied for foreign bodies measuring between 2 and 5 to 6 mm.
- For cases involving an oroantral communication or fistula, or foreign bodies measuring 2 cm or less, a fistula repair approach was performed as described by Sabatino et al.
- The Caldwell-Luc approach was selected in cases without an oro-antral fistula and for foreign bodies larger than 2 cm, especially when the endoscopic approach failed.

We adopted a “wait-and-see” approach for patients with foreign bodies smaller than 1 to 2 mm, monitoring them without immediate surgical intervention when clinically appropriate.

## 5 Results

A total of 165 patients with suspected sinonasal pathology were consecutively evaluated in the study. To define the primary analytical cohort, patients were categorized based on clinical and radiological findings:

- 111 patients exhibited sinusitis without evidence of a foreign body;
- 6 patients had a maxillary foreign body without associated sinusitis;
- 48 patients presented with both odontogenic sinusitis and a maxillary foreign body.

The male-to-female ratio was 1.01, corresponding to 83 males and 82 females.

Regarding the disease chronology, 129 patients exhibited a chronic form of sinusitis, defined as persisting for more than three months since its onset, whereas 26 patients presented with the acute form. Foreign bodies were identified in 6 patients without associated sinusitis.

We also sought to highlight the presence of an oroantral fistula in these patients.

Specifically, out of the total cohort, 73 patients exhibited an oroantral defect associated with the foreign body, whereas 92 patients did not present with any oroantral breach.

According to Felisati's classification, 8 cases were identified as a pre-implantological complication, 37 as implantological complications, and 115 as classical complications. Different surgical approaches were performed: 29 patients received surgery entirely through a nasal approach, 37 were managed exclusively through an oral approach, and 95 received simultaneous combined treatment via oral and nasal routes.

Obstruction of the ostiomeatal complex was observed in 77 cases.

The analysis focused primarily on patients with sinusitis associated with a foreign body. Within the study population of 165 patients, 48 were identified as having a foreign body associated with the sinus pathology. Among these, 32 patients (67%) underwent a combined surgical approach, with endoscopic nasal and oral access performed in the same operative session. A total of 12 patients (25%) were treated with an exclusively oral surgical approach, while 4 patients (8.33%) underwent surgery via an exclusively endoscopic nasal approach (Figure 5).

**Figure 5:**
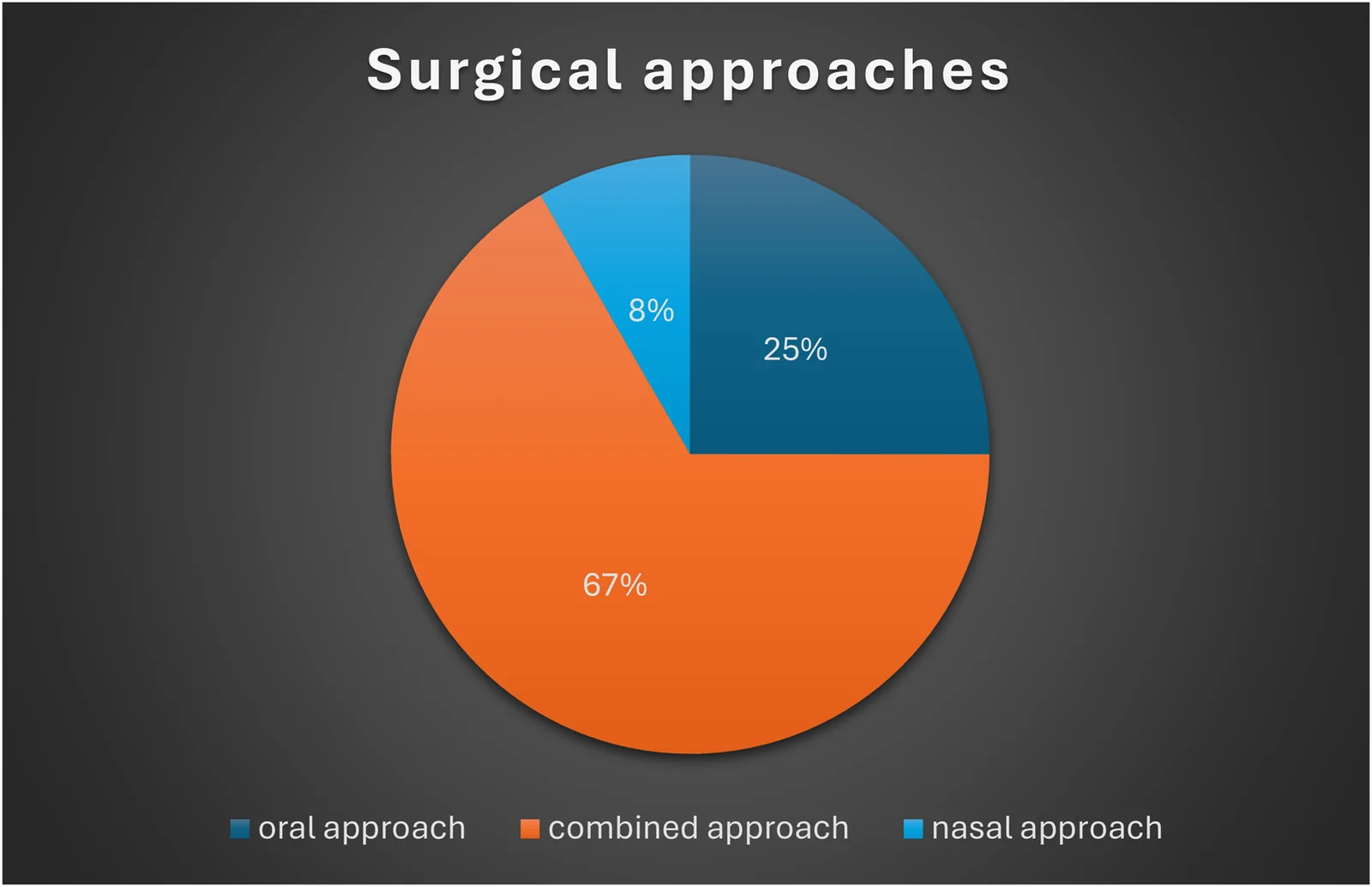
Surgical approaches in our study population.

No major complications occurred, and no revision surgeries were required. Complete resolution of symptoms and closure of the oroantral fistula were achieved in all included patients over a mean follow-up period of 6 months (minimum 3 months).

## 6 Discussion

In this study, we aimed to categorize patients based on their clinical presentation, commonly associated conditions, specific etiologies, and corresponding treatments, with particular emphasis on the therapeutic management of patients presenting with foreign bodies. Due to the lack of established guidelines, there is a pressing need to develop standardized classification criteria for this condition. There is still no consensus on the optimal management of foreign bodies within the maxillary sinus. Surgical removal is recommended when the foreign material is responsible for, or contributes to, the development of sinusitis. Given the diverse clinical presentations and the necessity of combined medical and surgical interventions, a multidisciplinary approach is essential when odontogenic sinusitis is suspected.

In such cases, the collaboration and cooperation between oral surgeons and otolaryngologists play a critical role in both clinical decision-making and operative management to ensure optimal patient outcomes.

Due to the complexity and variability of such cases, we implemented a dedicated treatment strategy aimed at the effective removal of these foreign bodies to resolve the associated odontogenic sinusitis and optimize clinical outcomes.

In the literature, some authors have suggested strategies for the surgical management of odontogenic conditions, highlighting the importance of a multidisciplinary surgical approach. Fadda et al. recently proposed the use of a combined oral and sinonasal approach in the following situations: (i) removal of infected dental implants with apical portions protruding into the maxillary sinus or other migrated materials, (ii) removal of infected grafting material, which may be difficult to clear using endoscopic access alone, (iii) management of foreign bodies or odontogenic cysts, (iv) treatment of periapical odontogenic infections, (v) management of maxillary osteitis or osteomyelitis, (vi) treatment of dentigerous cysts, (vii) complications related to dental extractions, and (viii) closure of oroantral communications (20).

Felisati et al. reported a prospective series of 257 patients with odontogenic sinusitis, proposing a therapeutic algorithm tailored to the underlying odontogenic cause, achieving a 98.8% success rate. Key recommendations include surgical maxillary sinus intervention via endoscopic sinus surgery (ESS) or Caldwell-Luc in all cases; implant/foreign body material removal in post-insertion infections; and surgical closure, such as with local flaps for oroantral communications (21).

The treatment algorithm presented in this study was developed retrospectively based on our clinical experience from 2020 to 2025. It adapts and expands upon the foundational frameworks previously proposed by González-García et al. and Brescia et al. The primary novelty of our proposed algorithm lies in the explicit combination of multiple concurrent variables: the status of the ostiomeatal complex (OMC), the precise dimensions and location of the foreign body, and the presence or absence of an OAC/OAF. By integrating these specific parameters, our algorithm provides a more detailed, stepwise rationale for selecting a simultaneous combined approach, which differs from previous algorithms that predominantly favor single-modality approaches.

Muammer Melih Sabhin et al. conducted a retrospective analysis of 94 patients treated using a combined approach involving Functional Endoscopic Sinus Surgery (FESS) and Caldwell–Luc procedures, utilizing two variations: radical antrostomy (RA) and mini-canine fossa antrostomy (MA). Their findings highlighted that in otorhinolaryngology practice, Caldwell–Luc surgery, whether through MA or RA, is primarily indicated to facilitate better access to the maxillary sinus in cases where endoscopic sinus surgery alone is insufficient (22).

A prospective study conducted by Petr Kocum et al. demonstrated that the one-stage combined ENT and dental approach was used in 94% of cases (groups 1 and 2) out of the total (364 patients) suffering from odontogenic sinusitis (ODS) with a success rate of 97% (23).

Endoscopic surgery was initially planned for foreign body removal, with a left uncinectomy and middle meatal antrostomy performed under general anesthesia. However, when endoscopic extraction proved unfeasible, the procedure was converted to an external approach using the Caldwell–Luc technique to allow improved visualization and access to the maxillary sinus (24). The intraoral approach offers the advantage of facilitating direct removal of the foreign body and concurrent closure of the OAC/OAF. However, it does not adequately address obstruction of the natural sinus ostium or the associated sinusitis. An intranasal approach, generally performed as endoscopic sinus surgery (ESS), is regarded as an effective, minimally invasive technique for implant retrieval, but removal becomes demanding when implants are situated in the anterior or inferior parts of the maxillary sinus. In anatomically difficult sites that are not easily accessible endoscopically, the Caldwell–Luc procedure represents a reasonable alternative (25). Among the intraoral approaches, the modified lateral window technique is frequently performed in order to treat conditions involving foreign body removal. The modified lateral sinus lift technique for root retrieval from the maxillary sinus offers several advantages: it shortens the duration of surgery, minimizes patient discomfort, necessitates less specialized surgical equipment, thereby making it accessible to dentists lacking endoscopic expertise, and helps preserve the alveolar bone, which supports subsequent implant placement and restorations (26). This technique enables a second-stage sinus graft to be performed through a lateral approach similar to that used in a maxillary sinus without prior treatment, thereby facilitating implant-supported prosthetic restoration (27).

In our cohort, the oral approach consisted of a modified mini Caldwell–Luc procedure with a mini canine fossa antrostomy. The Caldwell–Luc approach is currently reserved for situations in which wider access to the maxillary sinus is required, such as the removal of sizeable foreign bodies.

In our experience the foreign body can change its position, so it is mandatory to perform a CT scan preoperatively; its position can also change the surgical planning, so the presence of both an ENT specialist and a dentist is advised. The location of the foreign body also warrants a multidisciplinary approach. When the foreign body is situated in a lower region of the maxillary sinus, transnasal removal becomes challenging; therefore, the presence of the oral surgeon in the operating room is essential to ensure safe and effective management.

It has therefore emerged that the lack of standardized protocols still necessitates ongoing efforts to identify the most appropriate surgical approach.

However, in our study, a multidisciplinary strategy proved to be predominant, taking advantage of the combined use of an oral approach, according to our standardized intraoral techniques and an endoscopic nasal approach. Follow-up in this cohort was primarily based on clinical symptom resolution rather than systematic long-term radiologic assessment or formal patient-reported outcome measures. Despite this limitation, the relatively large number of patients with maxillary sinus foreign bodies and the consistent use of a predefined treatment algorithm yield findings that are directly applicable to routine clinical practice. Further prospective or multicenter studies are needed to confirm and refine these criteria and to better identify which patient subgroups derive the greatest benefit from a simultaneous combined approach.

## 7 Conclusion

The results obtained in our study population, in which 67% of patients with foreign bodies were treated with a simultaneous combined surgical approach (endoscopic and oral), clearly indicate that this strategy was the most frequently utilized method in our cohort and appeared effective for foreign body management in appropriately selected patients.

This integrated technique overcomes the anatomical and technical limitations of each individual access, providing wider visualization and more comprehensive access to the maxillary sinus cavity, thereby allowing a more precise and complete removal of the foreign body and reducing the risk of recurrence or complications.

According to our experience, the simultaneous combined approach therefore represents a new frontier in the management of foreign bodies in patients suffering from odontogenic sinusitis.

## Data Availability

The raw data supporting the conclusions of this article will be made available by the authors, without undue reservation.
